# Parry-Romberg Syndrome: A Report of a Rare Case and a Comprehensive Review

**DOI:** 10.7759/cureus.67345

**Published:** 2024-08-20

**Authors:** Harikrishnan Marappan, Raja AM

**Affiliations:** 1 Ophthalmology, Karuna Institute of Medical Sciences, Palakkad, IND; 2 Ophthalmology, All India Institute of Medical Sciences, Madurai, IND

**Keywords:** management, facial asymmetry, neurocutaneous disorder, progressive hemifacial atrophy, parry-romberg syndrome

## Abstract

Parry-Romberg syndrome (PRS), also recognized as progressive hemifacial atrophy (PHA), is a rare medical condition affecting the dermis, subcutaneous tissue, and occasionally underlying anatomical structures such as muscles and bones. While the etiology of this condition remains incompletely elucidated, it has been hypothesized that trauma, autoimmunity, infection, and autonomic dysregulation may constitute potential contributory factors. Typically, the onset of symptoms occurs within the initial two decades of life, though instances of late-onset PRS manifesting in the sixth and seventh decades of life have also been documented. The disorder is distinguished by a gradual progression over two to 20 years, ultimately culminating in stabilization.

The local manifestations of PRS are accompanied by systemic symptoms. Common neurological complications include seizures and headaches. Due to the rarity of PRS, there are no established guidelines for imaging, treatment, and follow-up. Therefore, management is tailored to each case, with treatment options primarily addressing symptoms.

## Introduction

Parry-Romberg syndrome (PRS), also known as idiopathic hemifacial atrophy or progressive facial hemiatrophy (PFH), is a rare condition characterized by progressive unilateral facial atrophy. Its historical roots can be traced back to ancient Egypt, as formally documented by Caleb Hillier Parry and later by Moritz Heinrich Romberg [1].

The incidence of PRS ranges from 0.3 to 2.5 cases per 100,000 population annually, with a male-to-female ratio of 1 to 3 [2]. It typically develops during the first two decades of life, with the average age of diagnosis being 13.2 years. This early onset underscores the importance of early detection and intervention, particularly in males, who tend to be diagnosed even earlier [3].

The exact cause of PRS remains unclear, but it is posited to involve autoimmune, genetic, and neurovascular factors. The primary effects target the skin and subcutaneous tissue and may impact musculoskeletal and central nervous system structures. They culminate in disfiguring facial changes and complications affecting bone and soft tissue integrity [4].

If left untreated, PRS can progress to a burn-out phase within two to 20 years of active disease activity. This progression often leads to pronounced facial asymmetry and disfigurement. Ocular manifestations, from uveitis to keratitis to retinal pathologies, require ongoing management. Recurrent episodes of uveitis or retinal vasculitis may result in secondary glaucoma with varying visual prognoses. Neurological manifestations, while often well-managed, can result in minimal disability, underscoring the severity of the condition and the need for ongoing management [5].

## Case presentation

**Figure 1:**
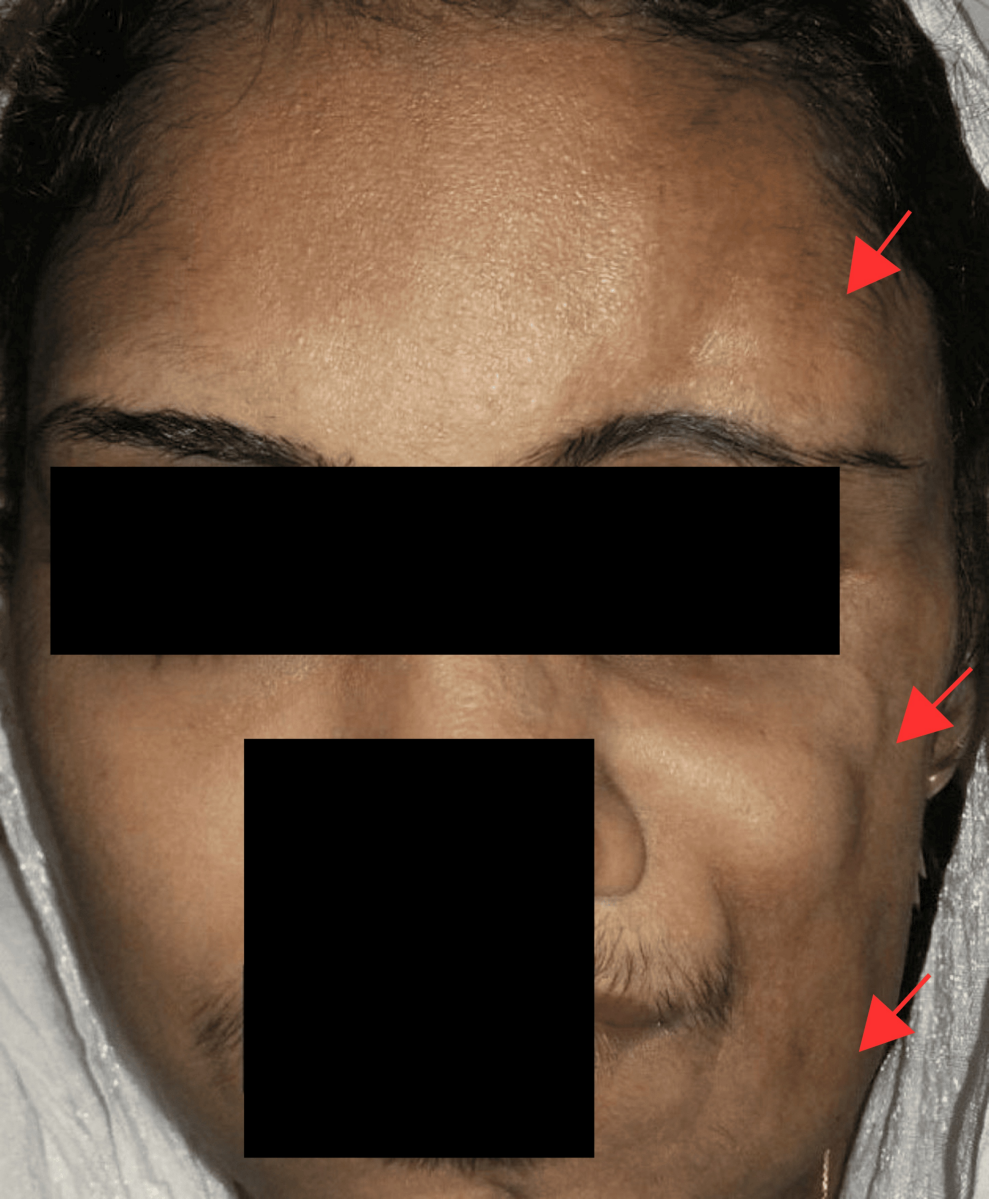
A patient diagnosed with PRS exhibits hemifacial atrophy on the left side of the face, including the forehead, cheek, and chin (red arrows), as well as 2mm of enophthalmos on the left side. PRS: Parry-Romberg syndrome

During a regular eye checkup at our ophthalmology clinic, a 28-year-old woman mentioned that she had been noticing gradual changes in her facial appearance and intermittent headaches over the past five years. She described the thinning of the skin and soft tissues on the left side of her face, which had led to a noticeable decrease in the size of her left cheek and temple. Notably, the patient had no history of physical trauma, systemic illness, or family history of similar conditions. Upon physical examination, our team observed hemifacial atrophy on the left side of her face, affecting her forehead, cheek, and chin. The skin on the affected area appeared thin and atrophic, with a visible loss of subcutaneous tissue. There were no signs of facial nerve involvement or other neurological deficits. The ophthalmic assessment utilizing Hertel's exophthalmometer revealed a 2mm enophthalmos on the left side, while the remaining ophthalmic evaluations were within the normal range (Figure 1).

Diagnostic workup

Based on our evaluation of the patient's clinical symptoms, we suspected PRS. We conducted extensive laboratory investigations, including a complete blood count, inflammatory markers, an autoimmune panel, and serological tests for infectious diseases. The results of these tests, all within normal limits, provided reassurance that there were no systemic causes for the observed facial atrophy. However, the patient chose not to pursue further investigations.

Management

In light of the progressive nature of PRS and the absence of definitive cures, patient management primarily centers on addressing cosmetic concerns and functional limitations. The patient received comprehensive counseling regarding the condition's natural progression and the crucial significance of regular monitoring to identify potential complications. Despite this guidance, the patient chose not to pursue any further treatment.

## Discussion

Parry-Romberg syndrome is a rare medical condition with a wide range of clinical symptoms, which can make it challenging to diagnose and treat. While it can impact individuals of any gender, it is more commonly seen in females [4]. It is important to note that this disorder typically manifests during the first two decades of life, although there have been documented cases of the disease occurring later in life [5,6]. It progresses slowly and often resolves in two to 10 years before stabilizing [7]. The specific cause of the disease remains unknown, although several theories, including autoimmune and neurovascular explanations, have been proposed [8,9].

This medical condition is characterized by progressive hemifacial atrophy of the skin and craniofacial tissue, typically affecting the dermatomes innervated by one or more branches of the fifth cranial nerve [7,10]. It leads to a concave facial appearance due to the atrophy of subcutaneous adipose tissue and muscular and osteocartilaginous structures [7]. While cutaneous involvement is limited, the condition may extend to the tongue, gingiva, teeth, and palate [8,11]. It commonly affects the ocular region, resulting in enophthalmos, pseudoptosis, uveitis, pupillary disturbances, heterochromia, and restrictive strabismus. The principal ocular manifestation is enophthalmos, arising from the depletion of adipose tissue surrounding the affected orbit [12,13]. In our case, the patient showed a 2 mm enophthalmos on the left side.

This syndrome is associated with various neurological complications, including trigeminal neuralgia, facial paresthesia, migraines, and seizures. Contralateral epilepsy is a rare occurrence. In our patient's case, a headache was the only observed clinical manifestation. Notably, characteristic features of PRS include tongue atrophy on the affected side, deviation of the mouth and nose toward the affected side, atrophy of the upper lip leading to teeth exposure, and small teeth with short roots on the affected side [14-16]. However, these features were absent in our patient.

As per Guerrerosantos et al. [16], PRS can be categorized into four types based on facial soft tissue depletion severity. Types 1 and 2 involve a reduction in facial soft tissue, whereas types 3 and 4 involve thinning of soft tissue and bone and cartilage atrophy. Type 1 represents the mildest form, while type 4 signifies the most severe and critical form. In this instance, our patient has been diagnosed with type 3 PRS, indicating a reduction and thinning of subcutaneous soft tissue on the left side of the face.

The patient demonstrates left-sided facial asymmetry characterized by hemiatrophy, a prominent left forehead with bony ridges due to loss of fatty tissue beneath the skin, 2mm enophthalmos on the left side, minimal pigmentation over the conjunctiva, and minimal hair loss. These findings are consistent with the classic features of PRS as outlined in previous literature [16]. The patient's neurological and ocular findings are within normal limits, except for enophthalmos, and the patient exhibits normal psychomotor functions.

Effective management of PRS necessitates a multidisciplinary approach involving physicians, surgeons, dentists, and psychologists due to the absence of a definitive cure. For PRS types 1 and 2, fat and dermis grafts are deemed the most suitable treatment. Additionally, pan-facial volumization with autologous fat cartilage and bone graft-galea flap injection effectively replenishes volume and restores contour to the aging face in PRS types 3 and 4 [13,16]. Despite the availability of treatment options, the patient has chosen not to undergo any intervention. Regular and long-term follow-up is imperative to monitor disease progression and optimize treatment outcomes.

## Conclusions

Parry-Romberg syndrome, also known as progressive hemifacial atrophy, is a rare neurocutaneous disorder characterized by the gradual degeneration of tissues on one side of the face. The complexity of its symptoms presents significant challenges in diagnosis and management, highlighting the need for a comprehensive, multidisciplinary approach involving dermatologists, neurologists, plastic surgeons, and other specialists. Despite ongoing research, the exact underlying causes of PRS remain unknown, emphasizing the critical need for extensive investigations to unravel its pathophysiology. More precise and effective treatment modalities can be developed by gaining a deeper understanding of the syndrome's mechanisms, ultimately improving the quality of life for individuals affected by this rare disorder. Continued research is essential for advancing innovative therapeutic interventions and enhancing healthcare outcomes for those grappling with PRS.
